# “A dream come true”: Perspectives on long-acting injectable antiretroviral therapy among female sex workers living with HIV from the Dominican Republic and Tanzania

**DOI:** 10.1371/journal.pone.0234666

**Published:** 2020-06-12

**Authors:** Deanna Kerrigan, Tahilin Sanchez Karver, Ohvia Muraleetharan, Virginia Savage, Jessie Mbwambo, Yeycy Donastorg, Samuel Likindikoki, Martha Perez, Hoisex Gomez, Andrea Mantsios, Miranda Murray, S. Wilson Beckham, Wendy Davis, Noya Galai, Clare Barrington

**Affiliations:** 1 Department of Sociology, American University, Washington, DC, United States of America; 2 Department of Health, Behavior and Society, Johns Hopkins Bloomberg School of Public Health, Baltimore, Maryland, United States of America; 3 Department of Health Policy, Yale University, New Haven, Connecticut, United States of America; 4 Department of Health Behavior, University of North Carolina, Chapel Hill, North Carolina, United States of America; 5 Department of Psychiatry, Muhimbili University of Health and Allied Sciences, Dar es Salaam, Tanzania; 6 HIV Vaccine Trials Research Unit, Instituto Dermatológico y Cirugía de la Piel, Santo Domingo, Dominican Republic; 7 Public Health Innovation and Action, New York, New York, United States of America; 8 Health Analytics and Outcomes, London, United Kingdom; 9 Department of Statistics, University of Haifa, Mt Carmel, Israel; University of Ghana College of Health Sciences, GHANA

## Abstract

**Background:**

Long-acting injectable antiretroviral therapy (LA ART) was found to be non-inferior to daily oral ART in Phase 3 clinical trials. LA ART may offer an important alternative for people living with HIV with challenges adhering to daily oral ART or preferences for non-pill-based regimens.

**Methods:**

Using a mixed methods approach integrating survey, in-depth interview and biological data from female sex workers (FSW) living with HIV in Tanzania (N = 208) and the Dominican Republic (DR) (N = 201), we assessed factors associated with the potential likelihood of LA ART use if it were available. We conducted multivariate logistic regression and thematic content analysis.

**Results:**

Likelihood of LA ART use was high with 84.92% of FSW from the DR and 92.27% of FSW from Tanzania reporting they would be “likely” or “very likely” to use LA ART if available (p = 0.02). In Tanzania better HIV-related patient-provider communication (AOR 4.58; 95% CI 1.90–11.05) and quality of HIV clinical care (AOR 3.68; 95% CI 1.05–12.86) were positively associated with the high likelihood of LA ART use. In the DR, easier clinic access was associated with a higher likelihood of LA ART use (AOR 3.04; 95% CI 1.41–6.56), as was greater monthly income from sex work (AOR 2.37; 95% CI 1.27–4.41). In both settings, years on ART was significantly associated with a strong likelihood of LA ART use (TZ: AOR 1.16 per year; 95% CI 1.00–1.34/DR: AOR 1.07 per year; 95% CI 1.00–1.14). Qualitative findings underscored enthusiasm for LA ART and reinforced its potential to address sex work-specific barriers to daily oral ART adherence including work-related schedules and substance use.

**Conclusions:**

We found a high likelihood of LA ART use if available among FSW in two diverse settings and documented barriers to future uptake. Community-driven approaches which include tailored health education and improved patient-provider communication and quality of care, as well as strategies to facilitate appointment adherence are needed to optimize LA ART use among FSW.

## Introduction

Long-acting injectable antiretroviral therapy (LA ART) has been found to be non-inferior to daily oral ART in Phase 3 clinical trials [1–6] and is poised to soon enter routine clinical care. This treatment modality has the potential to address many barriers to daily oral ART adherence among people living with HIV (PLHIV) and as a result has critical implications for both individual health and broader HIV transmission dynamics [7]. Studies carried out among PLHIV receiving LA ART in the context of clinical trials have found that the majority of participants preferred LA over daily oral ART and reported significantly higher satisfaction with LA versus oral ART [8–12]. Research on the acceptability of LA ART among PLHIV conducted outside of trials where participants were receiving LA ART, also demonstrates significant interest in this potential treatment option [13–15, 16].

In these prior studies, many PLHIV have characterized LA ART as simpler, easier and more convenient than daily oral ART [8, 9, 11, 12, 50] and described it as increasing a sense of freedom and normalcy in their daily lives [8, 11, 13, 50]. PLHIV have often reported that LA ART reduced experiences of internalized stigma associated with the ongoing reminder of living with HIV linked to daily pills and had the potential for less discrimination than oral ART as it was considered more confidential [8, 12, 13]. A minority of participants in prior research have expressed concerns about the potential for stigma associated with being seen by others in the clinic, particularly in the case that LA ART would necessitate more frequent visits appointments to receive injections, compared to their current frequency of clinic attendance to manage the use of daily oral ART [11, 13, 15, 50].

In deciding whether to switch to LA or stay on oral ART, research has found that important factors considered by PLHIV include being reassured of the efficacy and side-effect profile of LA ART [11, 13, 14] and being able to manage potential injection site reactions (ISRs) [13, 15]. Patient reported outcomes (PRO) data from clinical trials have found that experiences of pain and discomfort decreased over time as they became accustomed to the injections, and most ISRs were mild or moderate [8–10]. Specific groups, including women living with HIV, have been found to express greater concern for ISRs [14, 15]. A few studies found that some groups are more likely to find LA ART acceptable, e.g. youth, those with lower ART adherence, and people who use drugs [13, 15].

The limited research on patient perspectives on LA ART is mostly from middle- and high-income countries and predominantly focused on men participating in clinical trials of LA ART [6, 8–10]. It is unclear if and how LA ART perspectives may differ in the context of lower resource settings or among populations experiencing multiple structural constraints to HIV care [10]. Very few LA ART studies have been conducted among key populations with higher HIV prevalence, greater stigma and discrimination, and poorer treatment outcomes [13, 14], and none of these included female sex workers (FSW). There is an urgent need to understand the perspectives of key populations living with HIV, such as FSW, who have lower ART adherence and viral suppression rates compared to women overall [17–19]. FSW could be among those best positioned to benefit from LA ART which may address some of the unique barriers that they experience in relation to daily oral ART use.

The purpose of this analysis was to assess interest in LA ART among FSW living with HIV and to identify factors that could facilitate or inhibit its future use as part of routine care, using a mixed methods approach. We explored these dynamics within an ongoing study of the social determinants of HIV, leveraging two existing cohorts of FSW in the Dominican Republic (DR) and Tanzania.

## Materials and methods

### Study design

The longitudinal observational study, “Stigma, cohesion and HIV outcomes among vulnerable women across epidemic settings” (R01MH110158), is being conducted with 410 FSW living with HIV in the DR (n = 201) and Tanzania (n = 208). The study integrates biologic, survey, and qualitative data to obtain a holistic understanding of the social determinants of HIV outcomes among FSW living with HIV across two distinct epidemic and geographic contexts. In both settings, women were eligible if they were 18 years or older, had a confirmed HIV-positive diagnosis and reported exchanging sex for money in the last month prior to their enrollment in the study. All participants were surveyed and had blood draws at 0-, 12- and 24-month intervals. A sub-sample of FSW at each site participated in qualitative interviews at each visit. The current analysis draws on baseline quantitative and qualitative data collected from 2017–2018. In both settings, existing HIV-positive cohorts of FSW were augmented and re-enrolled and consented into the current study. These cohorts allowed for assessment of interest in and population-specific barriers and facilitators to LA ART among FSW.

### Study sites and participants

The HIV epidemic in the DR is concentrated among key populations, including FSW [20, 21]. The most recent estimate of national HIV prevalence among FSW was 4.0% [22], which is nearly 6 times the 0.7% overall national adult prevalence [23]. We established a cohort of FSW living with HIV in the greater Santo Domingo area of the DR in 2011 as part of an implementation science project called *Abriendo Puertas* (Opening Doors) focused on improving HIV care and treatment outcomes. Initial recruitment took place predominantly by FSW peer navigators, complemented by recruitment of FSW living with HIV by other key informants, such as clinicians, and study participants.

In Tanzania, HIV prevalence is higher in the Iringa region (9.1%), where recruitment took place, compared to nationally (5%) [24]. We established a cohort of FSW in Iringa in 2015 for a community-randomized trial of a community-driven intervention Project *Shikamana* (Let’s Stick Together in Swahili) which sought to reduce HIV incidence and improve care continuum outcomes. In that study, we found a baseline HIV prevalence of 40.9% [25]. We used time location sampling (TLS) to recruit venue-based FSW to the cohort from two distinct communities in the Iringa region. TLS entailed identifying days and times when the target population gathered at sex work venues, constructing comprehensive sampling frames of venues and daytime units, then randomly selecting and visiting venues and daytime units, and systematically collecting information from eligible members of the target population during those periods.

### Data collection procedures and measures

All visits for the current study occurred in private offices at the Instituto Dermatológico y Cirugía de la Piel (IDCP) in Santo Domingo, DR or Muhimbili University of Health and Allied Sciences (MUHAS) offices in Iringa, Tanzania. The baseline survey examined demographic, behavioral and socio-structural factors related to HIV care outcomes among FSW. Participants provided blood for the purposes of viral load testing. The following measures were included in the structured survey assessment.

#### Perceptions of LA ART

The main outcome of interest was defined as a strong likelihood of using LA ART (very likely) versus lower degrees of likelihood of using LA ART (likely, unlikely and very unlikely) if available. We assessed perceptions and potential barriers to LA ART use including concerns related to monthly medical appointments at the clinic for injections, ease of getting to the clinic for appointments, and stigma related to LA ART.

#### HIV care and treatment variables

Participants were asked how long they had been living with HIV as well as the number of years they had been on ART. Survey questions also assessed current ART use, any prior treatment interruptions and adherence to daily oral ART in the last 4 days [26]. To assess HIV viral load from blood samples we utilized Polymerase chain reaction (PCR) technology with the Roche Amplicor HIV-1 Monitor Test. Viral suppression was defined here as <400 copies/mL.

#### Socio-structural factors

We assessed internalized HIV stigma and sex work stigma using established aggregate measures validated among PLHIV including FSW [27, 28]. We have used these measures in prior our work with FSW living with HIV in both the DR [29] and Tanzania [23] and have found they have strong reliability. HIV and sex work stigma consisted of aggregate measures with 4-point Likert scale answers ranging from strongly agree to strongly disagree. The HIV stigma scale consisted of 10 items and produced a high reliability in each country (alpha = 0.91 in DR vs. 0.88 in TZ). The sex work stigma scale consisted of 13 items and also produced a high reliability in each country (alpha = 0.85 in DR vs. 0.84 in TZ). ART stigma was not used as an aggregate measure, rather each statement served as an indicator assessing the level of ART stigma. Responses for ART stigma were dichotomized using “yes” versus “no/not sure”. We assessed experiences of gender-based violence (GBV) using an adapted version of the Conflict Tactics Scale [30], tailored to FSW [31].

We explored patient-provider communication using the Patient Reactions Assessment (PRA) scale on perceptions of and satisfaction with clinical care [32]. The PRA scale consisted of 15 items with responses captured using a 4-point Likert scale ranging from strongly agree to strongly disagree and produced a high reliability in each country (alpha = 0.90 in DR vs. 0.89 in TZ). Clinic dynamics were explored using three statements serving to assess the experiences of participants in the clinic environment. These questions included perceptions of being treated with respect by clinic staff and availability of clinic staff to help patients, with answers ranging from never to always, and overall satisfaction with the clinic, with answers ranging from weak to excellent. For the purposes of analysis, clinic dynamic measures were dichotomized to compare highest level versus all others.

#### Demographic variables

We assessed participants’ age; relationship status, number of children, income per month, including from sex work; as well as mobility and migration outside of their current city or town of residence, overall and for sex work. We also screened for alcohol and druse use in the context of sex work over the last 30 days.

### Statistical data analysis

We conducted descriptive analyses of the survey data, examining the frequency and distributions of socio-demographic and behavioral characteristics (counts, mean, median, range, etc.) for participating FSW across the settings. We examined the statistical differences in socio-demographic and behavioral characteristics by country using chi-square tests for categorical variables and two sample t tests or Wilcoxon rank sum tests for continuous measures. We later assessed the perspectives on LA ART across each setting through chi-square tests of association for those dichotomized variables. We conducted stratified bivariate and multivariate logistic regression analysis to identify factors associated with a strong likelihood of LA ART use. Marginally significant variables (p < .10) from the bivariate models were included in the initial multivariate regression model with backwards stepwise procedure applied to develop a final model. Variables remaining in the final multivariate models were those with p<0.05.

### Qualitative data collection and analysis

We established an embedded qualitative cohort of 40 women participating in the parent study described above (20 from each country cohort). We used stratified, purposeful sampling [33] to select approximately 20 women who were virally suppressed at baseline and 20 women who were not suppressed, in each setting. The first interview was used to explore the phenomenon of living with HIV, from diagnosis to present HIV care and treatment experience. We also included a module on views related to LA ART. We first described the concept of LA ART to participants relaying: “*Now*, *if there was the option of an HIV treatment that you would receive as an injection (in the backside) once a month or once every two months at a clinic*, *and it worked just as well as the daily pill to control your HIV (keep you suppressed)*, *how would you feel about that injectable HIV treatment option*?”. We then explored participants’ potential interest in this option compared to daily oral ART and possible barriers and facilitators to uptake.

All interviews were conducted in Spanish or Swahili by trained interviewers, audio-recorded, and transcribed. Data analysis was led by the first author and implemented by study team members, independent from the interviewers. We began our qualitative analysis by writing a narrative summary of each participant’s HIV story [34, 35]. We included a summary of responses to the questions on LA ART and reviewed these summaries to identify recurring and important themes. We systematically organized themes within each country and then compared across the settings. We integrated survey and interview findings to gain a more complete picture of FSW’s perspectives on LA ART [36].

### Ethical considerations

The study was approved by the Institutional Review Boards of the Johns Hopkins Bloomberg School of Public Health in the USA, the Instituto Dermatológico y Cirugía de la Piel in the DR, and Muhimbili University of Health and Allied Sciences in Tanzania. All participants provided informed consent and were compensated ~$USD5 per study visit.

## Results

### Quantitative findings

#### Socio-demographics and HIV outcomes of the samples

As seen in Table 1, the median age at baseline among FSW living with HIV was significantly higher in the DR at 40 years, versus 31 years in Tanzania. Women in the DR had been living with HIV for significantly longer on average (7.96 years) than in Tanzania (1.60 years). Substance use (either alcohol or drug use) during sex work in the last 30 days was more prevalent in the DR (77.50%) than in Tanzania (56.25%). All forms of stigma (HIV, sex work, and ART stigma) were greater among FSW in the DR than in Tanzania. GBV in the last 6 months was significantly lower (22.50%) in the DR versus 33.17% in Tanzania. Perceived quality of HIV clinical care was significantly lower among FSW in Tanzania versus the DR. In the DR, 72.6% of FSW were virally suppressed versus 48.5% in Tanzania. Reported adherence to daily oral ART in the last 4 days was similar among those on ART across settings (~83%).

**Table 1:** Sociodemographic characteristics and HIV outcomes among FSW living with HIV.

Variables	Dominican Republic	Tanzania	p-value
***Demographic characteristics and socio-structural factors***			
Age in years (median, range)	40 (18–66)	31 (19–60)	<0.001
Currently married or cohabitating	65/200 (32.50%)	48/208 (23.08%)	0.033
Median monthly income: > Median 10,000 Pesos DR/90,000 Shillings TZ	97/200 (48.50%)	103/208 (49.52%)	0.837
Median monthly income for sex work: > Median (6,000 Pesos DR/ 40,000 Shillings TZ	93/200 (46.50%)	100/208 (48.08%)	0.750
Number of children (median, range)	3 (0–9)	2 (0–7)	<0.001
Mobility outside residence area	56/200 (28.00%)	69/208 (33.17%)	0.257
Mobility specifically for sex work	36/200 (18.00%)	17/206 (8.25%)	0.004
Substance use during sex work	155/200 (77.50%)	117/208 (56.25%)	<0.001
Any GBV in the last 6 months	45/200 (22.50%)	69/208 (33.17%)	0.016
***Experiences with stigma***			
HIV Stigma (median, range)	23 (13–33)	16 (10–40)	<0.001
Sex Work Stigma (median, range)	35.5 (24–52)	36 (17–51)	0.373
*ART Stigma*:			
I don’t want people to see me take my HIV medicines	117/189 (61.90%)	136/194 (70.10%)	0.090
I am embarrassed to get my HIV medicines from a pharmacy	27/189 (14.29%)	45/194 (23.20%)	0.026
Taking my medicines reminds me I have HIV	116/189 (61.38%)	159/194 (81.96%)	<0.001
***Provider and clinic dynamics***			
Patient Provider Communication (PRA Scale) (median, range)	44 (34–60)	47 (24–60)	<0.001
The staff at the clinic where you get your HIV care is always available to help you	163/200 (81.50%)	90/122 (73.77%)	0.101
The staff at the clinic where you get your HIV care always treats you with respect	184/200 (92.00%)	94/122 (77.05%)	<0.001
Overall, the services at the clinic where you get your HIV care are excellent	57/200 (28.50%)	26/121 (21.49%)	0.164
***HIV outcomes***			
Years Living with HIV (median, range)	7.96 (0.12–37.99)	1.60 (0.07–19.67)	<0.001
Years on ART (median, range)	5.59 (0–21.68)	1.13 (0–19.67)	<0.001
Currently on ART	179/199 (89.95%)	148/194 (76.29%)	<0.001
ART adherence last 4 days	149/179 (83.24%)	124/148 (83.78%)	0.895
Virally suppressed	146/201 (72.64%)	99/204 (48.53%)	<0.001

#### Perspectives on LA ART use

As shown in Table 2, enthusiasm for LA versus daily oral ART was high. Over 90% of survey respondents in both countries reported that if given a choice they would prefer LA ART to daily pills. When asked about the likelihood that they would use LA ART if available, approximately 84.92% in the DR and 92.27% in Tanzania indicated that they would be “likely” or “very likely” to use LA ART (p = 0.02). Potential concerns associated with LA ART use among FSW living with HIV also varied per setting. Among FSW in the DR there was significantly greater concern than in Tanzania about getting to the clinic to receive monthly injections and potential stigma related to receiving monthly LA ART in clinics.

**Table 2:** Perspectives on LA ART among FSW Living with HIV in the DR and Tanzania.

Frequency (%) of key variables	DR (n = 199)	Tanzania (n = 194)	p-value
*Personal method preference*, *if given a choice*:			
Daily pill	9.55%	6.70%	0.302
Injection every month	90.45%	93.30%	
*Likelihood of using LA ART if it were available*:			
Unlikely/Very Unlikely	15.08%	7.73%	0.022
Likely/Very Likely	84.92%	92.27%	
*Concerned about going to monthly clinic appointments to receive LA ART*:			
Not at all concerned/A little concerned	62.81%	95.88%	<0.001
Somewhat/Very concerned	37.19%	4.12%	
*How easy would it be for you to get to the clinic every month to receive LA ART*:			
Not very easy/Not easy at all	20.60%	3.09%	<0.001
Somewhat/Very easy	79.40%	96.91%	
*How concerned about stigma related to monthly LA ART*:			
Not at all/A little concerned	83.42%	95.36%	<0.001
Somewhat/Very concerned	16.58%	4.64%	

#### Factors associated with the likelihood of using LA ART

Multivariate models developed per setting identified factors associated with a strong likelihood of LA ART use among FSW living with HIV (Table 3). In both settings, years of living with HIV remained significant (DR: AOR 1.07 per year, 95% CI 1.00–1.14 and TZ: AOR 1.16 per year, 95% CI 1.00–1.34). In the DR, specific factors associated with a greater odds of LA ART use included greater monthly income from sex work (2.37, 95% CI 1.27–4.41) as well as ease of getting to the clinic for injections (AOR 3.04, 95% CI 1.14–6.56). In Tanzania, these factors included higher ratings of HIV-related patient-provider communication (AOR 4.58, 95% CI 1.90–11.05) and greater perceived quality of clinical HIV care (AOR 3.68, 95% CI 1.05–12.86).

**Table 3:** Unadjusted and adjusted odds of strong likelihood to use LA ART among FSW living with HIV.

Country	Factors	OR	95% C.I.	AOR	95% C.I.
**Dominican Republic** (n = 189)	> Median monthly income	2.04	[1.15–3.62]**		
	> Median monthly income from sex work	2.51	[1.40–4.50]***	2.37	[1.27–4.41]***
	Years living with HIV	1.05	[1.00–1.10]*		
	Years on ART	1.08	[1.01–1.14]**	1.07	[1.00–1.14]**
	Above the median patient-provider communication score (PRA scale)	1.66	[0.94–2.93]*		
	*ART Stigma*: I don’t want people to see me take my HIV medicines	0.58	[0.32–1.07]*		
	*ART Stigma*: I am embarrassed to get my HIV medicines from a pharmacy	0.43	[0.19–0.98]**		
	*Clinic dynamics*: The staff at the clinic where you get your HIV care is always available to help you	2.06	[1.00–4.25]*		
	Somewhat/Very easy to get to clinic monthly for LA ART	2.62	[1.29–5.31]***	3.04	[1.41–6.56]***
	Somewhat/very concerned about stigma/discrimination related to LA ART	0.47	[0.22–1.01]*		
**Tanzania** (n = 109)	> Median monthly income	1.67	[0.93–2.99]*		
	Years living with HIV	1.08	[0.99–1.18]*		
	Years on ART	1.12	[1.00–1.24]**	1.16	[1.00–1.34]**
	Above the median patient-provider communication score (PRA scale)	6.45	[2.82–14.72]***	4.58	[1.90–11.05]***
	*ART Stigma*: I am embarrassed to get my HIV medicines from a pharmacy	1.85	[0.90–3.81]*		
	*Clinic dynamics*: The services at the clinic where you get your HIV care are excellent	4.11	[1.43–11.81]***	3.68	[1.05–12.86]**
	Somewhat/very concerned about stigma/discrimination related to LA ART	0.17	[0.04–0.86]**		
	Not ART adherent in the last 4 days	3.13	[1.10–8.91]**		
	Not virally suppressed	1.75	[0.97–3.16]*		

p-values:

*p<0.10;

**p<0.05;

***p<0.01

### Qualitative findings

Demographic characteristics of the qualitative subsample of FSW did not vary significantly from the parent cohort. However, the qualitative sample was stratified on viral suppression, whereas half of participants in each country were virally suppressed and half were not. As a result the qualitative sample reflects diverse experiences with engagement in care and ART adherence, allowing us to explore perspectives on LA ART among those with and without treatment challenges. Both participants who were suppressed and those who were not expressed favorable perceptions of LA ART in terms of its ability to address logistical and psychosocial barriers to adhering to daily oral ART and generally felt that potential barriers could be addressed, making it an appealing option.

#### Advantages and facilitators

In-depth interview participants from both countries expressed strong enthusiasm for LA ART as an alternative to daily oral ART. In the DR, participants referred to the possibility of LA ART as “*phenomenal*” and “*lifechanging*”. In Tanzania, similar positive language was used to describe LA ART, such as *“the injection method would be the best of all*,*”* when comparing it to daily oral ART. Most participants reported that they would prefer LA to daily oral ART and felt that the majority of other FSW living with HIV whom they knew would also be interested. A few women drew an analogy between LA ART and the ease and popularity of injectable contraception.

Many participants felt LA ART would provide relief from the onus of taking daily pills. Even among participants who reported years of medication adherence and viral suppression, taking oral ART every day was characterized as burdensome and inconvenient. Participants described forgetting to take daily ART as a formidable barrier to adherence that LA ART would mitigate. Participants often reported that taking oral ART added to their ongoing daily stress, which was already high. Participants noted that some women were becoming tired of taking pills after many years. While adherence to daily oral ART was viewed as requiring constant energy and dedication, appointments to receive LA ART were seen as more finite and more manageable. Women in both countries felt that receiving LA ART as a monthly or every other month injection would be easier to remember than taking daily oral ART pills. As seen in the quote below, women also noted that LA ART may alleviate stigma and discrimination because it is more private and confidential than daily pills.

*This would be a dream come true*. *I wouldn’t have to hide my pills*. *I wouldn’t have to be worried about losing track of time or worried that if I had to go out for work and I didn’t bring them*, *or that someone has found them*…*that [LA ART] would be perfect*.

-FSW from the DR, age 28

*I would receive [LA ART]*. *That is better*, *because you do not have the trouble of taking it every day*. *So*, *when you get a shot*, *you go back after a month to get another one*. *So*, *it would be better than the ones you take every day*. *That is why people get exhausted*, *because of taking them every day*. *But the monthly one*, *no one would become exhausted*.

-FSW from Tanzania, age 25

LA ART was also perceived as a potential solution for sex work-specific barriers related to adherence to daily oral ART. For example, some participants discussed LA ART as more compatible with their irregular and unpredictable work schedules, which often involved late nights and alcohol use. Often mobile and needing to spend multiple hours or days with clients with little notice, participants referenced experiences in which they had not brought sufficient pills for the time outside of their home or in which their planned time to take their medications conflicted with the time they were with clients.

*Definitely there is an advantage for women that are sex workers*…*If there is an injection that they can use*, *then they can drink [alcohol] and not have to worry about whether they took their pills or not…And there are cases when a colleague will call me and say that she left her pills at home and cannot take them…That wouldn’t happen with the injection*.

-FSW from the DR, age 41

*You know some of us go to work at a late hour*, *they get back from work late*, *and when they get home*, *they are already late*, *they’ve missed their time for pills*. *But when she gets an injection*, *she just memorizes the day to go for injection*, *she gets her injection*, *and continues with her work*.

-FSW from Tanzania, age 27

Additionally, many participants expressed the need to hide their pills from their clients and other people in their work, households, and communities to avoid stigma, discrimination, and potential violence. LA ART was viewed as helping to avoid those negative situations and outcomes, since no one could discover their HIV medicine.

*The act of leaving the room and going to shower*, *my client can possibly start checking my bag and can easily see the pills in my purse*… *But if I have been injected*, *will he be able to discover that*? *Who will know*? *So the injections are the best of all*.

-FSW from Tanzania, age 33

The issue of side effects was discussed in relation to both daily oral and LA ART. Multiple participants viewed LA ART as an option that might alleviate the negative side effects of daily pills, including stomach issues, nausea, drowsiness, and potential longer-term toxicities, while some participants did anticipate ISRs from LA ART. However, perhaps because these would be once a month or less, these potential side effects were described as more manageable than the daily side effects that some women experience with oral ART. LA ART was also found to be an appealing option for many participants who were suffering from food insecurity, as current daily oral medications caused them negative side effects when taken on an empty stomach.

#### Disadvantages and concerns

When asked about potential disadvantages to LA ART, many women did not see any. A minority in each setting expressed concerns around clinic attendance to receive injections including the fear that this may lead some people to assume that they were HIV-positive. This concern seemed to be dependent on how frequent clinic visits for injections may be (e.g. monthly, bimonthly, every 3 months). However, even when such a concern was voiced, it did not seem to overshadow the perceived advantages of LA ART or likelihood to use it if available as seen in the quote below.

*I think all of them will opt and love this* … *because when you are using pills*, *you can forget many times/we forget many times*. *Or maybe if they inject you*, *3 month dose*, *that will be good*, *so you will come to the hospital after 3 months*, *that will so good* … *When you go to the hospital after every month*, *this can make other people notice you are infected* … *injection will be good*, *how you are injected 3 months dose like when we do on contraceptive injection that works for three months*.

- FSW from Tanzania, age 31

Among the minority who did identify concerns with or disadvantages of LA ART, the main issues were the fear of and potential for ISRs. Additionally, a few participants perceived that some women could forget their clinic appointments. Cost and time associated with clinic visits were also mentioned as potential barriers.

*Maybe [a disadvantage is] if someone got the injection and then forgets the date to return to the clinic*. *So*, *in my opinion*, *people should be given*, *like*, *a calendar*. *So that someone won’t forget the dates and they should know that*, *‘on a certain date*, *I have to return for the injection*.*’ But the injection has more advantages*, *as you don’t get it every day and it will make people healthy*. *[But] someone will just get the injection and can forget*, *as can happen with the pills*.

-FSW from Tanzania, age 31

In the DR, a few additional concerns were raised. One participant argued that she would prefer her current oral medications over LA ART because, similar to injectable forms of birth control, the consequences of missing one shot were higher than the impact of missing one pill. Concerns over being able to trust health services, and, in turn, the injection itself, were raised by a minority of participants. An additional finding observed in a small number of interviews was that when presented with information about LA ART, some participants appeared to confuse the language and concepts related to injections for the purposes of HIV treatment with the idea of injections as a cure for HIV or an HIV vaccine, given the association between injections as antibiotic treatments or as vaccines. One Dominican participant stated:

I am someone who does not like pills. I take them because it is an obligation and needed, but if there were a vaccine that would provide the same efect and do the same job and allow me to maintain the same strength and vitality, then I would me so happy.

-FSW from the DR, age 31

Related to this dynamic, one DR participant worried that some FSW receiving LA ART might stop using condoms consistently, if they believed that they were cured of HIV, further highlighting the importance of clear communication and tailored health education and messaging related to LA ART.

## Discussion

This mixed methods study is one of the first in-depth examinations of the perspectives of FSW living with HIV from two distinct geographic and epidemic contexts regarding LA ART. Findings reveal that attitudes towards LA ART are overwhelmingly positive and highlight the need to tailor use of this biomedical innovation to the needs of specific groups such as FSW where daily oral ART challenges have been documented.

Quantitative results indicate strong interest in using LA ART if available across settings. Findings from multivariate modeling indicates that access and interactions with clinics will be salient issues and potential barriers or facilitators to the successful implementation and scale-up of LA ART depending on how they are managed. For example, in the DR we found that reporting more difficulties with clinic access were significantly associated with greater likelihood of using LA ART if available. In Tanzania, perceptions of better patient-provider communication and the quality of care were also significantly correlated with increased interest in LA ART in that setting, indicating the importance of supporting strong patient-provider communication in future interventions around LA ART decision-making. In turn both material access *getting to the clinic* (e.g. transport, distance, cost) [37, 38] and psychosocial dynamics *within the clinic* (e.g. how groups do or do not feel respected and well-treated) will both be important issues to attend to as LA ART rolls-out across distinct settings [37–41]. These factors are also known barriers to retention in care and adherence to daily oral ART,[42–44] suggesting that while LA ART will alleviate many challenges to adherence, there will still be ongoing and important issues to attend to in order to optimize HIV outcomes [45].

Qualitative findings provided additional insight into how LA ART may fit into the daily lives and lived experiences of FSW living HIV. Across the two countries, LA ART was seen as alleviating the psychosocial and logistical burden linked to daily pill taking and the potential for stigma associated with pills, as has been documented among PLHIV participating in clinical trials [8, 46]. In our qualitative work with this sample, we also documented the importance of LA ART to address structural factors such as food insecurity that are known to impede oral ART use [47–49]. Additionally, LA ART was understood to have the potential to address sex work-specific barriers to adherence including working late at night, erratic schedules, mobility, and alcohol use related to sex work. By providing an alternative treatment method that may overcome these and other barriers to treatment adherence for FSW, LA ART offers the possibility of impacting individual and population level HIV outcomes.

A minority of in-depth participants expressed concerns about side effects, including ISRs. Qualitative data reveals that although LA ART clinical trial participants reported injection-related side effects, most also described learning to manage these side effects to make them more tolerable, given the other benefits of LA ART [50]. Similarly, Patient Reported Outcomes (PRO) measures from the Phase 3 studies demonstrated an increase in the “acceptability” of pain associated with the injections over time with the majority of participants reporting the pain as “totally/very acceptable”[51, 52]. Educating prospective LA ART users among the FSW population about patient experiences can help alleviate some of these concerns.

Some FSW in the current study were also concerned about stigma associated with receiving injections and being “discovered” by others at the clinic, echoing concerns raised by participants in the clinical trials [8, 50] and nuanced by the fact the sex workers face multiple forms of intersecting stigma when attending health clinic appointments. While many PLHIV face this potential challenge currently in relation to accessing pill refills in many instances monthly but in other cases every few months and even up to six months, more frequent (monthly or bimonthly) injections at the clinic may be of additional concern [50]. This underscores the importance of continuing to challenge the underlying social and structural dynamics contributing to HIV-related and other forms stigma [53, 54]. Without doing so, these factors may ultimately limit the potential of injectable ART to fully reach and assist PLHIV, particularly those from already marginalized population groups. Additionally, these findings suggest the distinct models of delivering the injections (e.g. not just clinic, but also community drop-in-centers or home-based care) LA [55] will be important to explore.

Overall, the potential downsides of side effects and stigma associated with receiving injections were perceived as more manageable than daily oral ART-related concerns, leaving FSW feeling that LA ART was a more appealing option regardless. Findings from both the survey and in-depth interviews suggest the importance of facilitating clinic access and appointment reminders to ensure adherence to injection schedules, which has critical implications for individual clinical outcomes (e.g. viral suppression, resistance) and population transmission dynamics (undetectable = untransmissible) [56, 57]. It is imperative that support systems are in place to remind FSW of injection appointments (e.g. having designated staff for retention) and to ensure FSW can access the clinic (e.g. addressing sex work stigma from healthcare providers).

Future implementation science to design and evaluate interventions that address these perceived facilitators and concerns to LA ART use are needed to optimize the introduction of this novel treatment option into routine clinical care among key populations such as FSW living with HIV, particularly in resource-constrained settings. Similar to strategies previously proposed for addressing barriers to ART access for FSW [58], efforts to address challenges to implementing LA ART with FSW should focus on reducing stigma associated with HIV and sex work, providing tailored educational materials which share patient experiences on LA ART and speak directly to questions and concerns specific to FSW populations, and developing solutions for logistical challenges. This may include the use of mHealth interventions for appointment reminders [59], and dedicated staff time for appointment reminders and facilitating clinic access particularly in the case of mobile FSWs, given the relationship between mobility and lower retention in care [60, 61]. Such strategies should be considered within a broader community empowerment approach to HIV among FSW, which has been considered a best practice globally and shown effective in both the current study settings [62].

This study is not without limitations including the cross-sectional nature of the current analysis and the limited information and experience that participants had about LA ART when answering questions about their preferences and likelihood to use it if available. Despite these limitations, interest was high, and the study was able to identify barriers and facilitators to optimal use in a key population that may benefit from LA ART. As a preliminary study, these results shed light on perspectives on LA ART among a group in which it has never been studied however additional future research is needed to further explore socio-cultural constraints of FSW and other key populations in these countries as well as in other settings. Additionally, as longer-term assessments of both the socio-behavioral considerations and potential biomedical impacts of LA ART is conducted [63], it will be important to ensure that women, including FSW, living with HIV are including in these studies.

## Supporting information

S1 File (DOCX)
