# Supplementary material for: “A dream come true”: Perspectives on long-acting injectable antiretroviral therapy among female sex workers living with HIV from the Dominican Republic and Tanzania
Source: PLoS One. 2020 Jun 12;15(6):e0234666. doi: 10.1371/journal.pone.0234666 (PMC7292359; doi:10.1371/journal.pone.0234666)
Supplement: S1 File — (DOCX) [file pone.0234666.s001.docx]

**Stigma, cohesion and HIV outcomes**

*Guide for qualitative sub-cohort: interview #1*

Date:___________________________________

Location:_______________________________

PID:_____________________________________

**Introduction**

*Thank you for taking the time to speak with me today. We appreciate your participation in the Shikamana Cohort and are happy to be able to talk with you today about your ideas and experiences. We are interested in hearing about how you take care of your health and things that help you stay healthy and things that sometimes get in the way. In particular we are interested in talking about how you manage your HIV status and your ability to access HIV care services and take your medications.*

1. I’d like to start by getting to know you a little better. Can you tell me about who you live with and where you live?

- How do you feel in this living arrangement?

- How do you get along with the people you live with?

2. Who are the most important people in your life at this time? This can be people in your family, community and beyond. Let’s make a list of these people that are most important to you.

(Probe below for each person on the list)

- What is your relationship to this person?

- How do they support you?

- How do you support them?

3. Can you tell me about your current work/employment situation?

- What are all of the different things you to do make money?

- What kind of environment are you working in?

- How do you meet/connect with clients?

4. Are you connected to a group or organization of other sex workers? Tell me about that…

-What types of activities do you do with this group?

-What types of support do you receive?

-What challenges are there with this group?

5. Now I would like to hear about your health. In general, how do you feel in terms of your health?

- How healthy do you currently feel? Tell me more about how you feel…

6. What things help you to feel and stay healthy? Tell me more…

7. What things get in the way of you feeling and staying healthy?

8. Now I would like to hear about your experience with HIV. Can you tell me about when you found out that you were living with HIV? Tell me about when you were diagnosed.

- How did you feel?

- How did you react to your diagnosis?

9. Who have you told about your diagnosis?

10. How do you feel talking to people about HIV?

11. How was your health when you were diagnosed?

- How did you feel at this time?

12. Tell me about the process of starting to receive HIV care and treatment after your diagnosis.

- How long did it take after you were diagnosed for you to have your first appointment with a doctor (or other health professional) and start treatment?

- What do you remember feeling during this time about needing treatment for the rest of your life?

13. Coming back to the present/today, how you are doing managing your HIV care and treatment?

14. How do you know how you are doing in terms of managing your HIV?

- What helps you know how you are doing with your HIV?
- What information does your doctor (or other health professional) give you to help you know how you are doing with your HIV management?
- Does your HIV doctor know about your work as a sex worker? Tell me more about that…

15. Are you familiar with the concept of viral suppression or having an undetectable viral load? What does that mean to you?

- How do you know if you are suppressed or undetectable?

16. Do you know if you are currently virally suppressed (or have an undetectable viral load)?

***For those who say they are undetectable:***

17. How long have you been undetectable?

18. How have you been able to become virally suppressed? What helped you to become undetectable?

19. What things have helped you to be undetectable? Tell me more about those…

20. Have you ever not been undetectable Tell me about what was going on when you were not undetectable.

- When you were diagnosed, do you remember if your viral load was undetectable?
- Have you ever had a period since your diagnosis when you went from being undetectable to detectable? Tell me about this…

21. What makes it hard to be undetectable?

22. If you have to identify the most important thing to help you be undetectable, what would it be?

23. How have the people we talked about earlier helped you to be undetectable?

24. Was there ever a time when you stopped taking your HIV medicines? Tell me about that time…

- What was going on at this time?

- What affected your daily routine with your medications?

25. How common is it to stop taking medication among other female sex workers living with HIV that you know?

- What makes it hard for other female sex workers living with HIV to take their medication?

26. How does the experience with medication compare among women living with HIV who are sex workers and women who are not sex workers?

27. In general, do you think being a sex worker affects your viral load? How so?

- How do people who know you are a sex worker treat you?

- How do people in the clinic treat you?

***For who say they are not undetectable:***

28. Have you ever been undetectable? When was that?

29. Tell me from your perspective why you aren’t currently undetectable?

30. What keeps you from being undetectable?

- What makes it hard or complicated for you to be undetectable?

31. Have you ever stopped taking them for a period of time? Tell me about this time…

32. Have you ever not been very consistent in your daily medication routine? Tell me more about this….

33. How common is it to stop taking medication among other sex workers you know living with HIV?

34. And how common is it to stop taking medication among other women living with HIV who are not sex workers?

35. In general, do you think being a sex worker affects your viral load? How so?

- How do people who know you are a sex worker treat you?

- How do people in the clinic treat you?

36. What would it take for you to become virally suppressed in the next few months?

***For all participants:***

37. Now, if there was the option of an HIV treatment that you would receive as an injection (in the backside) once a month or once every two months at a clinic, and it worked just as well as the daily pill to control your HIV (keep you suppressed)…how would you feel about that injectable HIV treatment option?

- ¿What do you think of an injectable treatment compared to a daily pill?

38. What are the advantages of an injectable form of treatment from your view?

39. What are the disadvantages of an injectable form of treatment from your view?

40. What would other sex workers think about an injectable treatment? Tell me more about your thoughts on this…

***Mchezo component:***

*Before we finish up, I wanted to just ask you a few questions about your economic situation and the role of mchezo.*

1. Now I would like to know a little more about what you do to support yourself beyond your income alone. What other ways do you help cover your expenses?
   - Do you ever seek loans? Tell me about that…from whom? How often?
   - Do you participate in michezo? Tell me about that…
2. Tell me what your thoughts are on michezo…

- What perceptions do you have of michezo here in your community?
- What do you think are the positive aspects of participating in michezo?
- What do you think are the negative aspects of participating in michezo?

43. What have your experiences with michezo been in the past?

- 1. Tell me about a time you participated in a mchezo…what happened?

44. What are the reasons you would choose or not choose to join a mchezo?

- 1. Thinking about why you may have joined one in the past, why did it seem like a good idea?
  2. Why do you currently choose not to be in a mchezo?

*We have come to the end of my questions. Thank you for sharing your experiences with me. Before we end, do you have any questions for me? Or, is there anything else you would like to share? Thank you again for your time and insights.*
